# Dietary spinach reshapes the gut microbiome in an Apc-mutant genetic background: mechanistic insights from integrated multi-omics

**DOI:** 10.1080/19490976.2021.1972756

**Published:** 2021-09-08

**Authors:** Ying-Shiuan Chen, Jia Li, Rani Menon, Arul Jayaraman, Kyongbum Lee, Yun Huang, Wan Mohaiza Dashwood, Ke Zhang, Deqiang Sun, Roderick H. Dashwood

**Affiliations:** a Texas A&M Health, Houston, USA; b Department of Chemical Engineering, College of Engineering, Texas A&M University, College Station, USA; c Department of Chemical and Biological Engineering, Tufts University, Medford, USA; d Department of Translational Medical Sciences, Texas A&M College of Medicine, Houston, USA

**Keywords:** Microbiome, transcriptome, metabolome, anticancer mechanisms, spinach

## Abstract

Complex interrelationships govern the dynamic interactions between gut microbes, the host, and exogenous drivers of disease outcome. A multi-omics approach to cancer prevention by spinach (SPI) was pursued for the first time in the polyposis in rat colon (Pirc) model. SPI fed for 26 weeks (10% w/w, freeze-dried in the diet) exhibited significant antitumor efficacy and, in the Apc-mutant genetic background, β-catenin remained highly overexpressed in adenomatous polyps. However, in both wild type and Apc-mutant rats, increased gut microbiome diversity after SPI consumption coincided with reversal of taxonomic composition. Metagenomic prediction implicated linoleate and butanoate metabolism, tricarboxylic acid cycle, and pathways in cancer, which was supported by transcriptomic and metabolomic analyses. Thus, tumor suppression by SPI involved marked reshaping of the gut microbiome along with changes in host RNA-miRNA networks. When colon polyps were compared with matched normal-looking tissues via metabolomics, anticancer outcomes were linked to SPI-derived linoleate bioactives with known anti-inflammatory/ proapoptotic mechanisms, as well as *N*-aceto-2-hydroxybutanoate, consistent with altered butanoate metabolism stemming from increased α-diversity of the gut microbiome. In colon tumors from SPI-fed rats, L-glutamate and *N*-acetylneuraminate also were reduced, implicating altered mitochondrial energetics and cell surface glycans involved in oncogenic signaling networks and immune evasion. In conclusion, a multi-omics approach to cancer prevention by SPI provided mechanistic support for linoleate and butanoate metabolism, as well as tumor-associated changes in L-glutamate and *N*-acetylneuraminate. Additional factors, such as the fiber content, also warrant further investigation with a view to delaying colectomy and drug intervention in at-risk patients.

## Introduction

The gut microbiome is strongly implicated in host physiology and pathophysiology.^1–3^ For example, studies in germ-free models of colorectal cancer (CRC) revealed decreased bowel inflammation and tumor outcomes as compared with the corresponding animals under conventional housing conditions.^4,5^ Fecal microbiota transplantation was used successfully to treat recurrent *Clostridium difficile* infection,^6^ and provided benefit to patients with inflammatory bowel diseases, functional gastrointestinal disorders, and obesity.^7^

There is increasing interest in defining interventions that alter the gut microbiota for disease prevention and treatment. Epidemiological studies indicate that CRC is associated with low consumption of green vegetables and fiber, whereas intake of dark leafy vegetables is linked to decreased risk.^8^ However, little is known about how these dietary intakes influence the crosstalk between gut microbiota, host transcriptomics, and pathogenesis in the gastrointestinal tract.

Spinach (SPI) is a dark leafy green vegetable with a high chlorophyll content and other diverse bioactives.^9–11^ We employed an adenomatous polyposis coli (Apc)-mutant rat model^12–15^ to examine anticancer outcomes from dietary SPI consumption. The polyposis in rat colon (Pirc) model mimics disease progression in human familial adenomatous polyposis patients, involving spontaneous tumor development both in the colon and in the small intestine.^15,16^ The genetic model circumvents the need for carcinogen treatment, used previously with dietary SPI,^17^ and the burden of adenomatous polyps facilitates temporal tracking of tumor suppression via colonoscopy.^12,14,18^

We observed significant antitumor efficacy from dietary SPI consumption, and despite the Apc-mutant genetic background, β-catenin protein levels remained highly overexpressed in colon polyps. Subsequently, mechanisms were pursued linking gut microbiome to host multi-omic changes in fatty acid metabolism, the tricarboxylic acid (TCA) cycle, and pathways in cancer.

## Results

### Antitumor efficacy of dietary spinach in an Apc-mutant rat model

**Figure 1.**
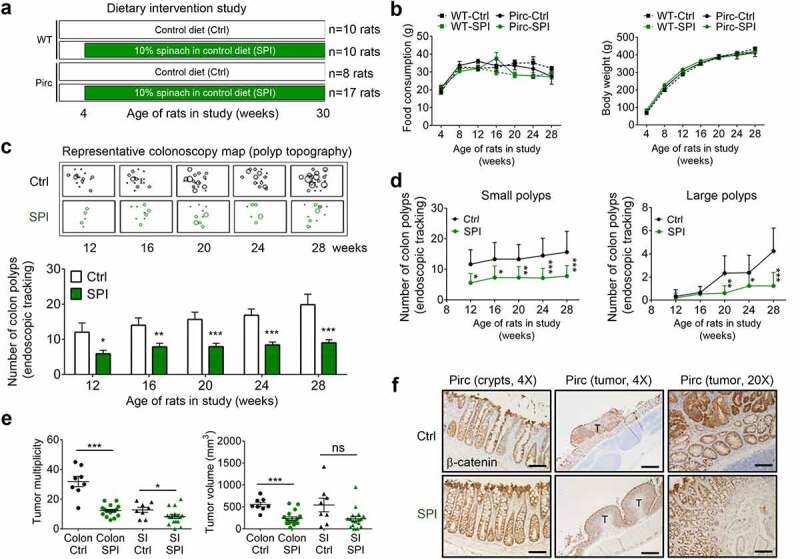
Dietary spinach suppresses tumor development in an Apc-mutant rat model. (a) Study design in Pirc and WT rats; n = 10 for WT-Ctrl (wild type rats given control/basal AIN diet) and WT-SPI (WT rats given AIN diet containing 10% freeze-dried spinach), n = 8 for Pirc-Ctrl, and n = 17 for Pirc-SPI. (b) Food consumption and body wt. gain. (c) Monthly endoscopic tracking of colon polyps in the Pirc model. The PLC classification system (Polyp number/Location/Clockwise orientation)^18^ was used to ascribe a unique address to every lesion in the colon, and ‘birds eye view’ topographic images were generated to illustrate the progressive changes observed in polyp size and location, along with bar graphs representing average multiplicity assessed via endoscopy. (d) Number of small (size grade 1–3) and large polyps (size grade 4–5) by monthly endoscopy, based on the PLC classification system.^18^ (e) Final tumor outcomes for colon and small intestine (SI) polyps at the 30-wk necropsy. (f) Immunohistochemistry staining of β-catenin in Pirc rats given Ctrl or SPI diets. Scale bar, 100 μm for 20X and 500 μm for 4X magnification. Numerical data are presented as mean±SEM; *p < .05, **p < .01, ***p < .001, ns statistically non-significant

Pirc and wild-type (WT) rats were fed AIN93 control (Ctrl) diet, or AIN93 diet containing 10% w/w freeze-dried baby SPI, starting at 4 weeks of age (Figure 1a). No significant treatment-related effects were observed with respect to food consumption and body weight throughout the study (Figure 1b). A ‘birds eye view’ topographical map of the colon was generated, representing each observable adenomatous polyp and its relative size, based on the PLC classification system (Polyp number/Location/Clockwise orientation)^18^ that ascribes a unique address to every lesion detected during monthly colonoscopy in the Pirc model. This approach provided early insights into antitumor efficacy, with suppression of adenomatous polyps occurring as early as 12 weeks into the experiment (Figure 1c), *i.e*., after 8 weeks of SPI treatment. During this period and at later times, colonoscopy data revealed consistent inhibition of small colon polyps, and significant suppression of large colon polyps after week 20 (Figure 1d).

When the experiment was terminated, after the rats had reached 30 weeks of age, tumor multiplicity was decreased significantly both in the colon and in the small intestine, and tumor volume also was reduced significantly in the colon by SPI treatment (Figure 1e). No marked changes were observed histologically, but bromodeoxyuridine (BrdU) labeling indicated reduced cell proliferation rates by SPI in some regions of the colonic crypt (Supplemental Figure S1). Immunohistochemistry and immunoblotting experiments revealed that β-catenin overexpression in colon tumors was unaffected by SPI treatment (Figure 1f and Supplemental Figure S2). Thus, despite the Apc-mutant genetic background, antitumor mechanisms other than β-catenin downregulation were pursued.

### The gut microbiota is altered by dietary spinach

**Figure 2.**
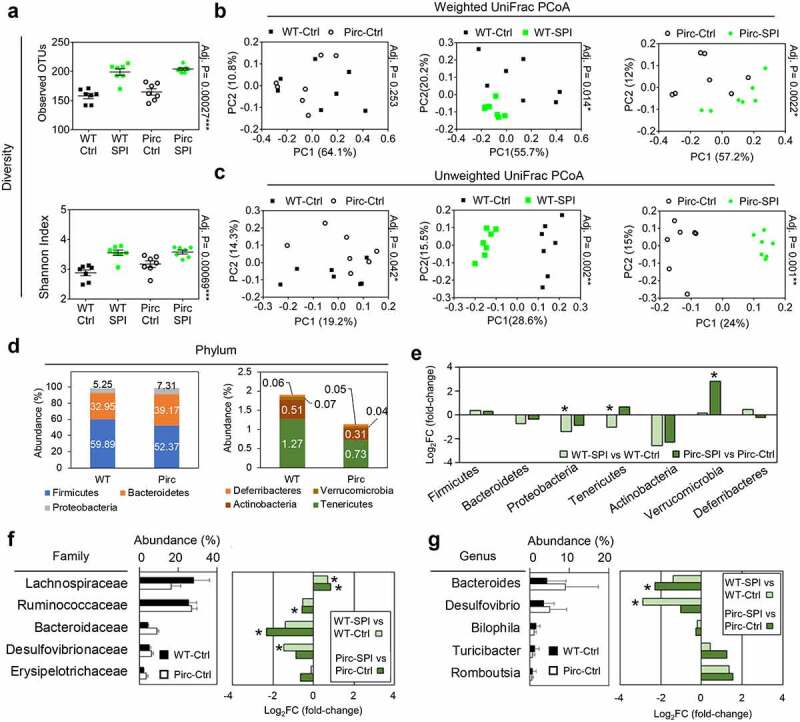
Microbial composition in relation to host genotype and dietary intervention. (a) α-diversity measured by observed OTUs and Shannon index; n = 7 replicates for 16S sequencing in each group. (b) Weighted and (c) unweighted UniFrac principal coordinate analysis (PCoA) plots displaying β-diversity. (d) Taxonomic composition in Pirc and WT rats at the phylum level. (e) Relative abundance changes at the phylum level after SPI intake. (f) Left, top five-most abundant family members. Right, abundance changes by SPI in Pirc and WT rats. (g) Left, top five most abundant genera. Right, abundance changes by SPI in Pirc and WT rats. Adjusted *p* values were calculated by Kruskal-Wallis test (*p < .05). For SPI-mediated changes in Family and Genus datasets, *p < .05. PERMANOVA analyses confirmed the overall conclusions, see text

We performed 16S rRNA sequencing of the gut microbial community in Pirc and WT rats. For a complete view of the taxonomic and other data, refer to Supplemental Tables S1–11. The observed Operational Taxonomic Units (OTUs, Supplemental Table S1) and Shannon index revealed that α-diversity was unaffected by host genotype but was increased significantly by SPI treatment in Pirc and WT rats (Figure 2a, black *vs*. green symbols). There was no segregation between Pirc and WT rats for weighted UniFrac principal coordinate analysis (PCoA) (Figure 2b), but a significant separation was observed in unweighted UniFrac PCoA (Figure 2c). The gut microbiome in both genotypes clustered separately in weighted UniFrac PCoA between Ctrl and SPI groups (Figure 2b), with a marked shift in unweighted UniFrac PCoA (Figure 2c). Applying additional permutational multivariate analysis of variance (PERMANOVA) to PCoA results generated the following data: WS *vs*. WC unweighted UniFrac *p* < .0001; PS *vs*. PC unweighted UniFrac *p* < .0001; WS *vs*. WC weighted UniFrac *p* < .0001; PS *vs*. PC weighted UniFrac *p* < .005. Collectively, these findings are consistent with previous work indicating that diet plays a dominant role over genetic background with respect to shaping interindividual variations in host-associated microbial communities.^19,20^

The Pirc model had a higher abundance of Bacteroidetes and Proteobacteria than WT rats, while Firmicutes, Actinobacteria, and Tenericutes were lower (Figure 2d), as observed in mouse and human microbiomes.^21–24^ Based on fold-change in abundance, SPI intake decreased Proteobacteria and Tenericutes, and increased Verrucomicrobia (Figure 2e). Changes also were noted at the Family (Figure 2f) and genus level (Figure 2g). For example, in Pirc and/or WT rats, SPI treatment increased the relative abundance of *Lachnospiraceae* and decreased *Ruminococcaceae, Bacteroidaceae*, and *Desulfovibrionaceae* (Figure 2f, green bars), and at the genus-level SPI ingestion reduced the relative abundance of *Bacteroides* and *Desulfovibrio* (Figure 2g, green bars). These results suggested that SPI consumption reshapes the microbiome composition, reversing the effects of the Apc-mutant background and host genetic predisposition.

**Figure 3.**
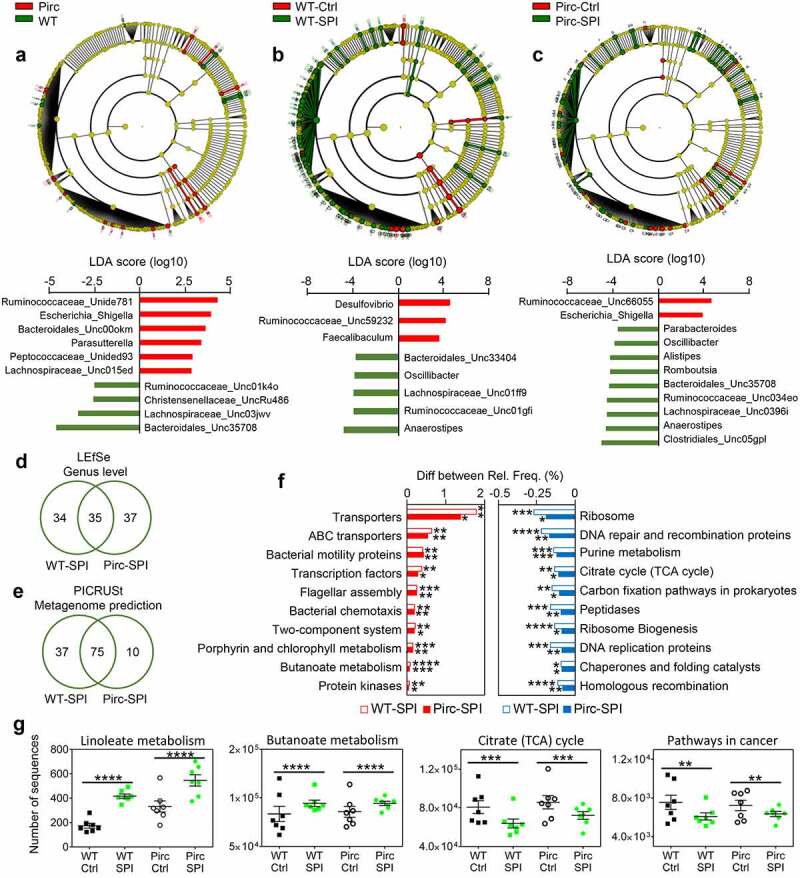
Dietary spinach reshapes the microbiome and its predicted metagenome. Cladograms and LDA scores for enriched clades in two-group comparisons of LEfSe data: (a) WT *vs*. Pirc (no SPI treatment); (b) WT±SPI; (c) Pirc±SPI. Cladograms illustrate all significant changes in taxonomy, whereas the bar graphs below show representative enriched bacteria taxonomy. Venn diagrams of (d) LEfSe data for enriched clades at the genus level and (e) metagenome prediction from PICRUSt in Pirc and WT rats after SPI treatment. (f) Prediction of top ten functional metagenomes in Pirc and WT rats given SPI (*p < .05, **p < .01, ***p < .001, ****p < .0001). (g) Linoleate (****p < .0001) and butanoate metabolism (****p < .0001) were increased significantly, whereas TCA cycle (***p < .001) and Pathways in Cancer (**p < .01) were decreased by SPI treatment in Pirc and WT rats. Statistical analyses were performed in STAMP with ANOVA. Supplemental Tables S2–5 provide complete LDA results, unclassified 16S rRNA seq data, and detailed PICRUSt information

Linear discriminant effect size (LEfSe) also was used to analyze OTU data (Supplemental Tables S2–4). From the corresponding cladograms (Figure 3a-c), host genotype and dietary SPI intake both influenced *Ruminococcaceae* and *Lachnospiraceae* family members. In response to SPI treatment, LEfSe analyses revealed that Pirc and WT rats shared ~50% commonality among changes at the genus level (Figure 3d). Phylogenetic Investigation of Communities by Reconstruction of Unobserved States (PICRUSt) categorized 328 terms following Kyoto Encyclopedia of Genes and Genomes (KEGG) analysis (Supplemental Table S5).

Linoleate and ether lipid metabolism were altered significantly in Pirc *vs*. WT rats, and PICRUSt revealed a marked effect of SPI intake. Thus, after SPI consumption, 85 terms (57 decreased and 28 increased) and 112 terms (71 decreased and 41 increased) were changed in Pirc and WT rats, respectively, and 75 terms overlapped between the two genotypes, *i.e*., 54 decreased and 21 increased (Figure 3e). Increases in membrane transporters, cell motility, signal transduction, transcription, carbohydrate metabolism, and kinases were among the top 10 terms prioritized by KEGG analysis in Pirc and WT rats given SPI, along with porphyrin and chlorophyll metabolism (Figure 3f). Decreased terms were related to protein translation, replication/repair, and energy/nucleotide metabolism. Pathway changes that were highly significant included an increase in linoleate and butanoate metabolism, and a decrease in the TCA cycle and pathways in cancer (Figure 3g).

### Spinach consumption impacts key genes associated with pathogenesis

**Figure 4.**
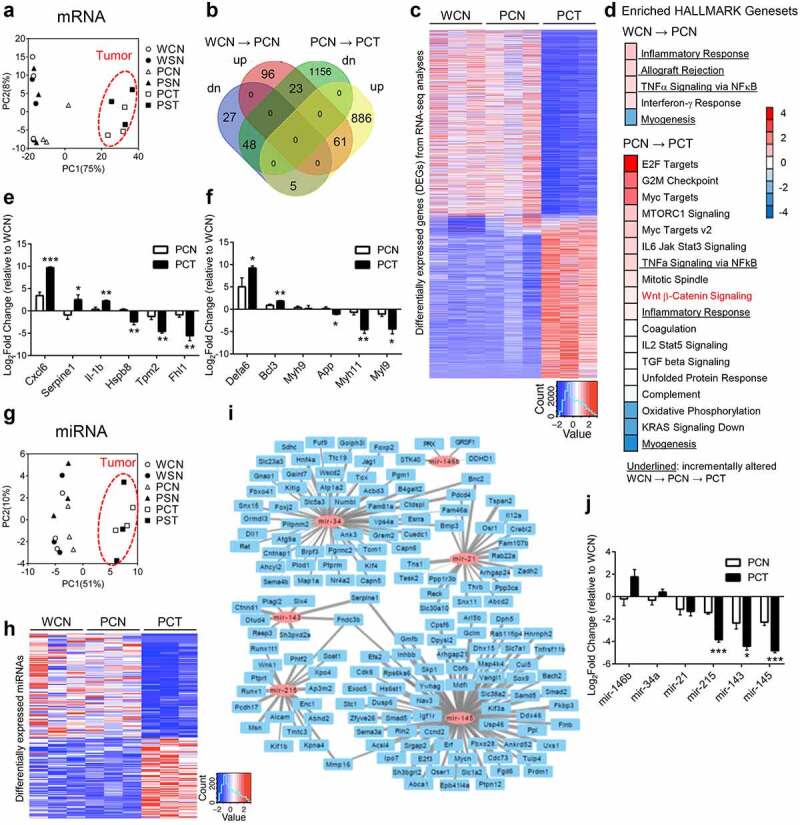
Transcriptomic analyses of colonic tissues obtained at 30-wks from Pirc and WT rats (no SPI treatment). (a) Principal component analysis (PCA) of RNA-seq data segregated tumor from non-tumor. WCN, WT-control diet-normal colonic mucosa; WSN, WT-SPI diet-normal colonic mucosa; PCN, Pirc-control diet-normal looking colonic mucosa; PCT, Pirc-control diet-colon tumor; PSN, Pirc-SPI diet-normal looking colonic mucosa; PST, Pirc-SPI diet-colon tumor. Each group contained three biological replicates, and Pirc normal looking colon was matched to Pirc colon tumor in the corresponding animal. (b) Venn diagrams summarizing differentially expressed genes (DEGs). (c) Heatmap of RNA-seq data representing all DEGs. For the corresponding categories that were prioritized, see (d). (**d**) Enriched GSEA HALLMARK analysis of DEGs between WCN and PCN (upper panel), and between PCN and PCT (lower panel). Underlined terms indicate pathways altered incrementally for group comparisons: WCN→PCN→PCT. Normalized enrichment score is indicated by color scale; −4 (blue) to +4 (red). Wnt signaling (red font) was one of several pathways enriched in the Apc-mutant model. (e) Validation by RT-qPCR of genes in five incrementally altered pathways, underlined in d). (f) Validation by RT-qPCR of genes related to tight junctions and antimicrobial function. (g) PCA of small RNA-seq data segregated tumor from non-tumor for miRNAs. (h) Heatmap of overall DEmiRs. For key miRNAs that were prioritized and validated by RT-qPCR, see panels (i) and (j). (**i**) Cytoscape view of conserved miRNA-RNA targets and the predicted negative associations between RNA-seq and small RNA-seq data. (**j**) Experimental validation of selected miRNAs in Pirc colon tumors. *p < .05, **p < .01, ***p < .001. Two-tailed *t*-test was performed for RT-qPCR results in two-group comparisons. Numerical data are shown as mean±SEM, n = 3

RNA-sequencing (RNA-seq) mapped 17,378 transcripts in colonic tissues from Pirc and WT rats, and principal component analysis (PCA) completely segregated tumor tissues from normal tissues (Figure 4a). Tumor development had a more marked effect on overall transcriptome levels than host genetics (Pirc *vs*. WT rats) and SPI consumption. There were 261 differentially expressed genes (DEGs) identified between Pirc and WT normal-looking tissues, half of which (138 genes) overlapped with 2180 DEGs associated with tumor development (Figure 4b). Heatmaps of all DEGs showed a distinct tumor feature when compared to Pirc and WT normal-looking tissues (Figure 4c, PCT *vs*. PCN and PCT *vs*. WCN). Beyond these tumor/normal comparisons, we were particularly interested in the specific gene changes that were prioritized in response to SPI treatment (Supplemental Figure S3), as discussed below.

Geneset Enrichment Analysis (GSEA) combined with HALLMARK identified five pathways altered significantly in Pirc normal-looking colon compared to WT normal colon (Figure 4d, upper panel), indicating differences at the level of host genetics. Three of these pathways were further altered in colon tumors (Figure 4d, lower panel, underlined), *i.e*., Inflammatory Response, TNFα signaling via NFκB, and Myogenesis. As expected in the Apc-mutant background, Wnt/β-catenin signaling was upregulated in Pirc colon tumors (Figure 4d, red font). Overexpression of β-catenin protein was associated with poly(ADP-ribose)polymerase (PARP) cleavage, increased cyclin D1, and decreased p53 in Pirc tumors compared to adjacent normal and WT normal colonic tissues (Supplemental Figure S2).

Other pathways of note were related to cell cycle changes, immune response, oxidative stress, and metabolism (Figure 4d). Using RT-qPCR for validation, genes upregulated significantly in Pirc colon tumors *vs*. adjacent normal-looking colon included *Cxcl6, Serpine1*, and *Il-1b*, whereas *Hspb8, Tpm2* and *Fhl1* were downregulated (Figure 4e). Compared to adjacent normal colon, upregulation of *Defa6* and *Bcl3* and downregulation of *App, Myh11*, and *Myl9* indicated changes in tight junctions and anti-microbial activity in Pirc colon tumors (Figure 4f).

We also mapped 559 microRNAs (miRNAs) via small RNA-seq, which segregated tumor *vs*. normal-looking colon (Figure 4g). Similar to the mRNA profiles (Figure 4c), miRNAs had a distinct tumor feature as compared to Pirc and WT normal-looking tissues (Figure 4h, PCT *vs*. PCN and PCT *vs*. WCN). There were 115 differentially expressed miRNAs (DEmiRs) associated with tumor formation (Figure 4h and Supplemental Table S6). We combined TargetScan with RNA-seq and small RNA-seq datasets to identify miRNA-RNA pairs most altered in the Apc-mutant background (Figure 4i). Validation by qPCR corroborated significant downregulation in Pirc colon tumors of miR-215, miR-143, and mir-145 compared with normal-looking colonic mucosa (Figure 4j). Other candidates, such as mir-34a and mir-21, did not reach statistical significance.

**Figure 5.**
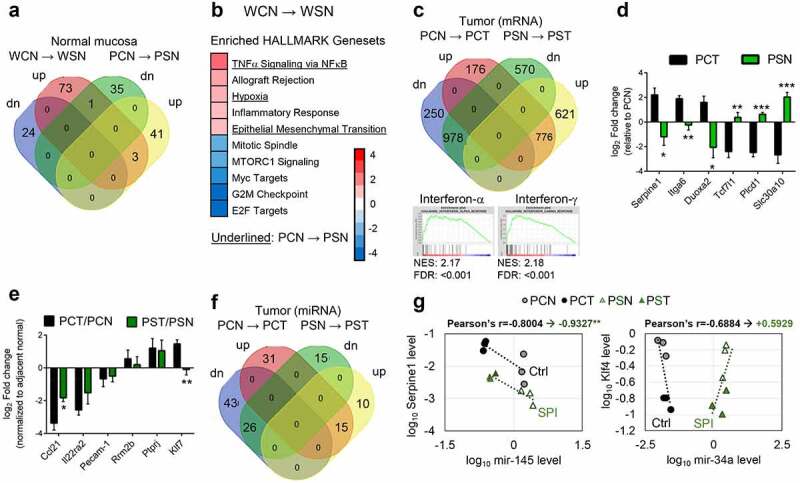
Transcriptome changes due to SPI treatment. (a) Venn diagrams of DEGs for miRNAs altered by SPI treatment in Pirc and WT rats. (b) Statistically significant pathways from GSEA HALLMARK analysis, highlighting SPI effects on WT normal colon. Underlined terms indicate affected pathways in Pirc normal looking colon. (c) Venn diagram of tumor DEGs, comparing Pirc colon tumors after Ctrl or SPI diet consumption, and (below) GSEA enrichment in tumors arising despite SPI treatment (putative ‘SPI-resistant’ colon polyps). (d) Validation by RT-qPCR of genes reversed in PCT *vs*. PSN groups. (e) Assessment of normalized molecular targets linked to oncogenic outcomes in Pirc colon tumors following SPI consumption. (f) Venn diagram of tumor DEmiRs in Pirc tumors from rats given Ctrl or SPI diet. (g) Corroboration of miRNA-mRNA relationships by RT-qPCR, in Pirc tissues from Ctrl and SPI treatment groups. *p < .05, **p < .01, ***p < .001. Numerical data are presented as mean±SEM, n = 3. For Pearson’s correlation, degree of freedom (N-2) = 4 and the critical value = 0.917 (*p* = .01). Supplemental Table S7 provides miRNA-mRNA prediction data for miR145/*Serpine1* and mir-34/*Klf4.*

Attention shifted next to SPI effects on predicted targets (Figure 5 and Supplemental Figure S3). Compared to the AIN basal diet control group, SPI consumption altered 4 genes in common among the 101 DEGs in WT rats and 80 DEGs in Pirc normal colon (Figure 5a). GSEA indicated significant downregulation of cell cycle-related pathways and upregulation of immune-related pathways in Pirc and WT rats fed SPI, with three pathways in common among the genotypes (Figure 5b). The latter pathways included TNFα Signaling through NFκB, Hypoxia, and Epithelial Mesenchymal Transition. After SPI consumption, 2945 DEGs were identified in Pirc colon tumors compared to normal colon, and 1754 of the DEGs also were detected in tumors *vs*. normal colon from rats given control diet (Figure 5c). Among the pathways most strongly implicated were IFN-α and IFN-γ for tumors from SPI-fed rats compared to rats given control diet.

We also considered two scenarios for the antitumor efficacy: (1) genes up- or downregulated in colon tumors relative to adjacent normal colon that were reversed by SPI in Pirc normal colon, and (2) genes that were normalized in colon tumors from rats given SPI compared with colon tumors from Pirc rats given control diet. The first scenario would implicate primary prevention of colonic aberrant crypt foci or microadenomas, before they advanced to later stages. These genes included *Serpine1, Itga6, Duoxa2, Tcf7l1, Plcd1* and *Slc30a10* (Figure 5d). Comparing tumor to tumor in scenario 2, *Ccl21* and *Klf7* were normalized by SPI ingestion, relative to basal diet (Figure 5e).

In terms of miRNAs, among 66 DEmiRs in colon tumors from SPI-fed rats, 41 DEmiRs similarly were detected in colon tumors from animals on Ctrl diet (Figure 5f). After investigating RNA-miRNA pairs and validating as before (Figure 4j), colon tumors exhibited loss of miR-145 with increased *Serpine1* and gain of mir-34a with reduced *Klf4* (Figure 5g and Supplemental Table S7). A negative correlation for mir-145/*Serpine1* was maintained after SPI consumption, whereas the mir-34a/*Klf4* trend was reversed by SPI treatment (Figure 5g).

### Crosstalk between microbiome and host transcriptome responses

**Figure 6.**
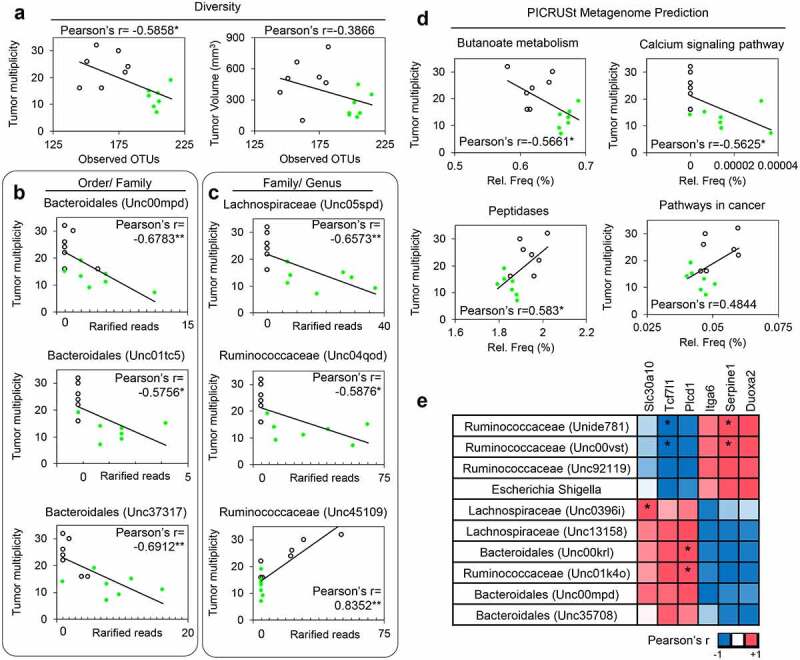
Integrating gut microbiome, gene expression and tumor outcomes after SPI treatment in the Pirc model. (a) Associations between gut microbiome diversity and host disease outcomes, expressed as tumor multiplicity or tumor volume. Associations also were probed between tumor multiplicity and the relative abundance of bacterial OTUs at the (b) family and (c) genus level. (d) Correlations between tumor multiplicity and metagenome prediction. (e) Associations between relative abundance of bacterial OTUs and anticancer/tumor suppressor genes identified in Pirc colon. Pearson’s correlation coefficient data were represented as positive (red) or negative (blue); asterisk indicates statistical significance. For Pearson’s correlation in a)-d), degree of freedom (N-2) = 12 and the critical value = 0.533 (*p* = .05), and 0.661 (*p* = .01). For Pearson’s correlation in e), degree of freedom (N-2) = 1 and the critical value = 0.997 (*p* = .05)

Integrating antitumor outcomes (Figure 1) with α-diversity (Figure 2), we observed a significant inverse association for tumor multiplicity (Figure 6a, left panel) but not tumor volume (Figure 6a, right panel). Tumor multiplicity was inversely correlated with three unclassified *Bacteroidales* families (Figure 6b and Supplemental Table S8). At the genus level, one unclassified *Lachnospiraceae* and one unclassified *Ruminococcaceae* genus were negatively correlated with tumor multiplicity, whereas one other unclassified *Ruminococcaceae* genus was positively correlated (Figure 6c). Metagenome prediction in relation to tumor multiplicity outcomes found significant inverse correlations for butanoate metabolism and calcium signaling, and positive associations for peptidases and pathways in cancer (Figure 6d).

We also compared microbiome and host gene expression changes based on the transcriptomic data (Figure 6e). Significant positive correlations were noted for *Lachnospiraceae* (Unc0396i) and the efflux transporter *Slc30a10, Bacteroidales* (Unc00krl) and *Ruminococcaceae* (Unc01k4o) and the phospholipase C family member *Plcd1*, and *Ruminococcaceae* (Unide781 and Unc00vst) and the serine protease inhibitor *Serpine1*. Negative correlations were detected for *Ruminococcaceae* (Unide781 and Unc00vst) and the transcription factor *Tcf7l1*.

### Metabolomic corroboration of mechanistic leads

**Figure 7.**
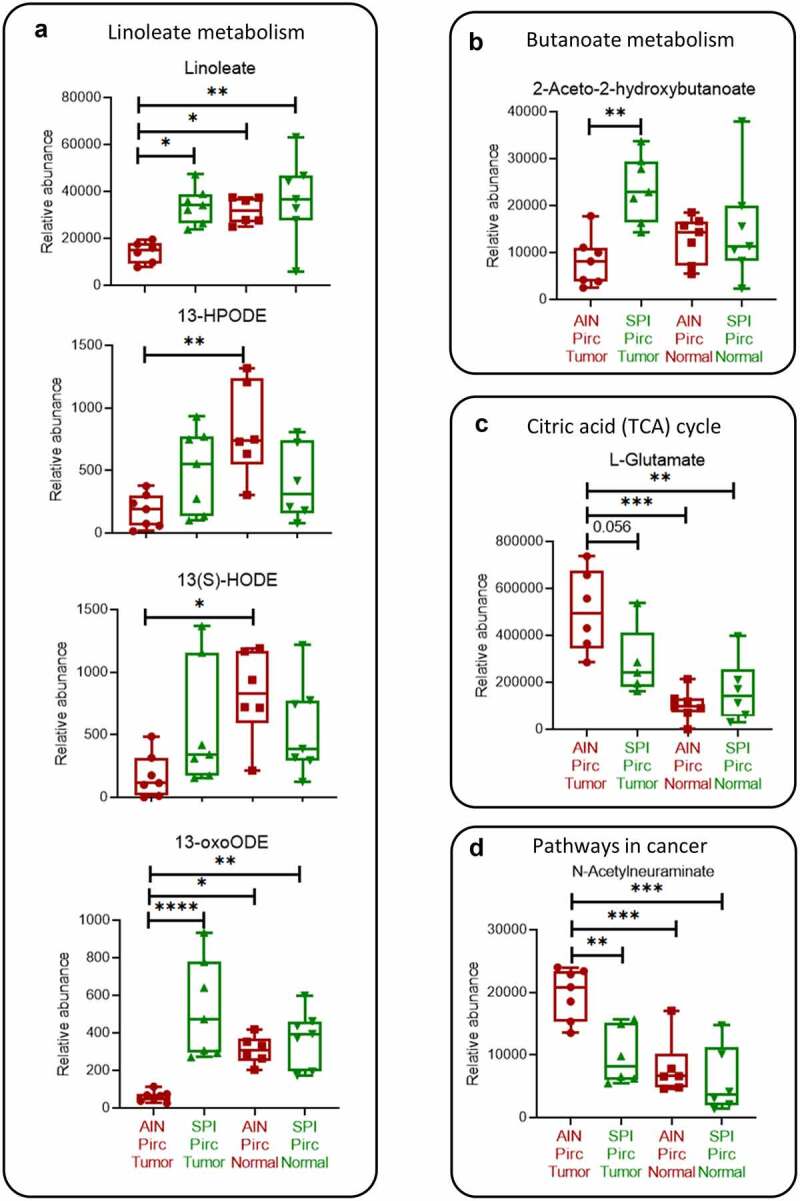
Metabolomic data after SPI consumption in the Pirc model. (a) Linoleate (linoleic acid) and its metabolites. (b) 2-aceto-2-hydroxybutanoate. (c) L-glutamate. (d) *N*-acetylneuraminate. Each datapoint represents one colon tumor or one normal colonic mucosa sample from rats in the corresponding groups. One-way ANOVA was used to compare the mean of each column with the mean of every other column, with Tukey correction for multiple comparisons (GraphPad Prism 9.0). *p < .05; **p < .01; ***p < .001; ****p < .0001

To validate correlations from the microbiome and transcriptome studies, metabolomics was performed on adenomatous colon polyps and normal colon tissues obtained from Pirc rats at 30 weeks (Figure 1a). As predicted from the microbiome data (Figure 3g), among the fifty-one metabolites identified in the rat (Supplemental Table S9) several were associated with fatty acid metabolism, the TCA cycle, and pathways in cancer (Figure 7a-d). Metabolites downstream of linoleate and 15-lipoxygenase-1 (15-LOX-1), such as (13S)-hydroxyoctadecadienoic acid (13(S)-HODE), exert proapoptotic antitumor mechanisms in CRC.^25–27^ Notably, lower levels of these metabolites in Pirc colon tumors tended to be normalized in adenomatous polyps following SPI treatment, comparable to the levels detected in normal-looking Pirc colon ±SPI (Figure 7a). Similar trends were observed for 2-aceto-2-hydroxybutanoate, which was increased significantly in colon tumors after SPI treatment (Figure 7b). On the other hand, L-glutamate and *N*-acetylneuraminate were detected at higher levels in colon tumors, and SPI treatment reduced these metabolites in adenomatous polyps, comparable to the levels observed in normal-looking Pirc colon ±SPI (Figure 7c and d).

## Discussion

We examined the interrelationships between host genetics, gut microbial composition, dietary exposure, and disease outcome in an Apc-mutant model that mimics hereditary human CRC.^12–15^ Lower gut microbial diversity was circumvented by feeding rats dietary SPI under conditions in which significant suppression of adenomatous polyps occurred in the colon and small intestine. This work extends prior observations on the decreased microbial diversity and loss of Firmicutes, *Clostridia*, and *Lachnospiraceae* in Apc^Min/+^ mouse^21^ and human colorectal cancers.^28,29^ In a short-term clinical study, Firmicutes was decreased by consumption of an animal-based diet, whereas Bacteroidetes was lowered by a plant-based diet.^20^ *Ruminococcaceae* phylotypes were increased by resistant starch in obese men, whereas *Lachnospiraceae* phylotypes were increased by non-starch polysaccharides.^30^ Phylum-, Family- and Genus-level taxonomic changes also were observed in the current investigation after intervention with dietary SPI, including increased Verrucomicrobia and *Lachnospiraceae* and decreased *Ruminococcaceae, Bacteroidaceae, Desulfovibrionaceae, Bacteroides*, and *Desulfovibrio*. The latter observations implicate a possible role for spinach in anti-inflammatory responses and enhanced gut barrier function.^31,32^

In contrast to the marked effect on the gut microbiome, long-term SPI intake exerted a relatively modest impact on host transcriptomics, based on mRNA and miRNA sequencing. We prioritized genes associated with adenomatous polyp suppression by SPI that were correlated with microbiome abundance. *Serpine1* encodes plasminogen activator inhibitor type 1 (PAI-1), which is elevated in sporadic and hereditary CRC, and has an essential role in extracellular matrix proteolysis and matrix metalloproteinase activity.^33–35^ Mucosal gene expression profiling of *SERPINE1*, plus inflammatory regulators such as *CXCL1, STAT3*, and *IL* family members, revealed associations with the decreased abundance of Firmicutes and Bacteroidetes subsets, as in human CRC.^36^ *DUOXA2* is a maturation factor for the epithelial antimicrobial dual oxidase DUOX2, which is among several NADPH oxidase/dual-oxidase family members deregulated in CRC and Crohn’s disease, acting via NFκB.^37,38^ *DUOX2* expression reportedly was negatively correlated with *Bacteroides, Lachnospiraceae*, and *Blautia*, but positively correlated with *Pasteurellaceae, Enterobacteriaceae*, and Gammaproteobacteria.^38^

We also extended our prior work on carcinogen-induced rat colon tumors that examined miRNAs and the mRNA targets.^17^ Mir-145 was among the most highly downregulated miRNAs in Pirc colon tumors, consistent with its proposed tumor suppressor role in human CRC, although mir-145 was unexpectedly upregulated in carcinogen-induced colon tumors,^17^ for reasons that remain unclear. Previous studies prioritized *SERPINE1* as a target of mir-143/145 in bladder cancer,^39^ as well as mir-34a in liver cancer, regulating *KLF4*.^40^ *KLF4* is targeted by multiple miRNAs,^40,41^ including mir-34a, and in the Pirc model the mir-34a/*Klf4* axis was altered by SPI treatment. *KLF4* is an important zinc-finger transcription factor involved in cell cycle regulation, somatic cell reprogramming, and tumorigenesis. Reduced *KLF4* is documented in rat^41,42^ and human colon tumors,^43,44^ suggesting an avenue for precision nutrition in the clinical setting.^45^

Metabolomic analyses corroborated several key findings from the microbiome and transcriptome studies, providing valuable insights into the anticancer effects of SPI. Tumor-associated linoleate and its 15-LOX-1-dependent intermediates were lower in Pirc controls fed basal AIN diet, and they were normalized in colon polyps after SPI treatment (Figure 7a), consistent with the proposed anticancer mechanisms of these metabolites.^25–27^ Several other intermediates associated with fatty acid metabolism also were detected in metabolomic analyses, such as 6-keto-prostaglandin E_1_ (6-keto-PGE_1_) and PGE_2_ ethanolamine; changes after SPI consumption were consistent with an overall shift toward anti-inflammatory, proapoptotic and tumor suppression pathways (Supplemental Figure S4). Thus, in SPI-fed rats, a decreased flux through pro-inflammatory leukotrienes and prostaglandins was paralleled by increased levels of 9-LOX, 15-LOX-1, prostacyclin, and cytochrome P450 (CYP) metabolites that are linked to anticancer outcomes.^25–27^

One noteworthy observation was that enzymes associated with fatty acid metabolism (Supplemental Figure S4, blue font) were not among the main candidates prioritized by RNA-seq analyses (Supplemental Figure S3). Why would the transcriptomic data following SPI treatment not implicate the enzymes involved in linoleate metabolism? We speculated that certain intermediates detected in rat tissues at 30 weeks (Figure 1a) might derive directly from the SPI incorporated into the AIN basal diet, and this was confirmed via unbiased metabolomic analyses of the freeze-dried spinach. Thus, among the 700+ analytes in SPI, several corresponded to key intermediates detected in Pirc colon tumors and normal-looking tissues (Supplemental Tables S9 and 10, green font). These included linoleate, 13(S)-hydroperoxy-9Z,11E-octadecadienoic acid (13-HPODE), and (9Z,11E)-13-oxooctadeca-9,11-dienoic acid (13-oxoODE), as well as L-glutamate and N-acetylneuraminate. Interestingly, the 2-aceto-2-hydroxybutanoate metabolite that was increased in colon tumors from SPI-fed rats (Figure 7b) was not detected in freeze-dried spinach, implicating beneficial butyrate-producing gut bacteria linked to increased α-diversity as the source.^46–48^

Although L-glutamate and *N*-acetylneuraminate were detected in freeze-dried spinach (Supplemental Table S10), colon tumors at 30 weeks had reduced rather than increased levels of these intermediates following dietary SPI administration (Figure 7c and d). Diminished L-glutamate levels in adenomatous polyps from SPI-fed rats would be synonymous with synthetic lethality,^49^ circumventing TCA cycle functions that are dependent on glutamine metabolism as a means of sustaining mitochondrial energetics. Lower levels of *N*-acetylneuraminate in tumors from SPI-fed rats would implicate altered cell surface glycans that are critical for pathways in cancer, including immune evasion, resistance to apoptosis, and enhanced proliferation, metastasis and angiogenesis.^50–52^ Notably, altered sialyation has been linked to activation of the inflammasome mediator eIF2,^50^ which in an APC-deficient background attenuates MYC-dependent apoptosis,^53^ unless circumvented by mechanisms that downregulate EIF2 – as observed for *Eif2b2* in colon tumors from SPI-fed rats (Supplemental Figure S3b, green arrow).

Limitations of the current investigation include the need for verification and quantification of metabolites using refence standards and NMR-based methodologies, and expanded metabolomic analyses beyond the predominantly hydrophilic analytes prioritized here, recognizing that chlorophylls and related phytochemicals can exert anticancer effects in the colon and other tissues.^9–11,54–61^ Freeze-dried SPI removes the water, leading to concentration of the constituent phytochemicals, such as betaine, chlorophylls, carotenoids, flavonoids, and polyunsaturated fatty acids, as well as the fiber content.^9–11^ There are many types of dietary fiber in commonly consumed foods, exerting differential impacts on the gut microbiota, short-chain fatty acid production, and metabolic regulation.^62–64^ Future studies should seek to corroborate the relative contributions of the fiber and phytochemical content, given the promising anticancer efficacy outcomes identified here. Clinical translation of freeze-dried whole foods, such as SPI, to at-risk patients might provide valuable quality-of-life benefits by delaying colectomy and drug intervention.^65^

In conclusion, we provided the first evidence for the marked anticancer efficacy of dietary SPI in the Apc-mutant Pirc model. After eliminating deregulated β-catenin as the primary mechanistic target, subsequent work identified significant reshaping of the gut microbiome in SPI-fed rats, along with changes in host transcriptomics and RNA-miRNA networks. Metabolomic analyses corroborated the predicted changes in linoleate and butanoate metabolism, TCA cycle, and pathways in cancer. Whereas butanoate metabolism was probably associated with increased α-diversity of the gut microbiome, multiple SPI-derived linoleate intermediates with known anti-inflammatory and proapoptotic mechanisms were detected at increased levels in the colon tumors from rats treated with dietary SPI. A recent report provided confirmation of 15-LOX-1/13(S)-HODE mechanisms using conditional knockout and over-expressing mouse models of colorectal cancer, which implicated LRP5-SNX17 interactions.^27^ Future experiments will seek to corroborate additional mechanistic leads from the work reported here, including the dietary fiber aspects and other targets identified by integrated multi-omics.

## Materials and methods

### Preclinical

Prior approval was obtained from the Institutional Animal Care and Use Committee. After weaning, Pirc (F344/NTac-*Apc^am1137^*, Taconic Farms, Inc. USA) and WT F344 male rats were assigned randomly to basal AIN93 control diet (Ctrl) or AIN93 diet containing 10% w/w freeze-dried baby SPI. Diets were replenished every 2 to 3 days, and animal body weights were monitored weekly. Monthly endoscopy was employed for temporal tracking of polyp development in the rat colon, as described before.^12,14^ Each polyp was assigned a unique ‘address’ in the colon, based on a reported methodology.^18^ Prior to termination, 3–4 rats in each group were injected with BrdU (100 mg/kg body weight), and animals were euthanized 1 h later by CO_2_ inhalation. A thorough necropsy examination was performed, and tissue samples were taken for histopathology and molecular analysis, as reported.^12,14^

### Proteins

Tissue sections (5 μm) were stained with hematoxylin and eosin (H&E), or immunostained with antibodies for β-catenin (BD Biosciences # 610153), cleaved caspase-3 (Cell signaling #9661), and BrdU (BD Biosciences # 347580), at the Research Histology, Pathology and Imaging Core, The University of Texas MD Anderson Cancer Center. A BrdU labeling index was determined, as described,^66^ and cleaved caspase-3 was quantified as percent positive-stained crypts in a given field. The corresponding β-catenin labeling index revealed no marked differences in Pirc rats ±SPI, including nuclear positivity within luminal, central and basal regions of the crypt column.^66^ Three biological replicates were employed for WT-Ctrl, WT-SPI, Pirc-Ctrl, and Pirc-SPI groups. For each tissue section, at least 15 independent fields were quantified in the proximal, middle, and distal regions of the colon. Immunoblotting was performed as reported,^12–14,67–70^ using Cell Signaling primary antibodies for PARP (#9542), β-catenin (#9581), p53 (#9282), and cyclin D1 (#2926), with β-actin as loading control (Sigma, A1978).

### Microbiome

Frozen gut contents from rats in each group were submitted for bacterial genomic DNA extraction at the Center for Metagenomics and Microbiome Research (CMMR), Baylor College of Medicine, Houston, Tx, followed by microbiome analyses as reported.^71–73^ The 16S rDNA V4 region was amplified and barcoded via PCR and sequenced using the MiSeq platform (Illumina) with the 2 × 250 bp paired-end protocol. OTUs at a similarity cutoff value of 97% were generated by the UPARSE algorithm and mapped to SILVA database. The dataset had a total raw read count of 1207636, with a minimum of 30714, a maximum of 48497, and a mean of 43556. For total mapped reads, the corresponding values were 261076, 7525, 10900 and 9324, respectively. OTU tables and Agile Toolkit for Incisive Microbial Analyses (ATIMA) were provided by CMMR for primary data visualization. ATIMA microbiome data were subjected to the Kruskal-Wallis test, with the adjusted *P*-values indicated in the figures. Beta diversity was assessed using the PCoA ordination on Bray-Curtis weighted and unweighted UniFrac distances to determine separation among or between groups. Weighted and unweighted UniFrac distances between groups were further analyzed by PERMANOVA using the R package. LEfSe used rarefied OTU data from CMMR, and results were generated with LDA>2 and *p* < .05 (Kruskal-Wallis test). PICRUSt used raw BIOM files to map Greengenes for metagenome prediction, coupled to Statistical Analysis of Metagenomic Profiles (STAMP). Results were replotted using Prism or Excel. To associate microbiome and disease outcome, bacterial diversity and OTUs were integrated with matched tumor multiplicity and tumor volume datasets. At the family and genus level, tumor outcomes were plotted by linear regression analysis, coupled to Pearson correlation coefficient. To integrate microbiome OTUs with host gene predisposition and anticancer outcomes, we calculated the Pearson correlation by group for enriched microbiome OTUs from LEfSe (genus level) and the reads per kilo base per million mapped reads (RPKM) of the host transcriptome. No additional FDR adjustment was made for the LEfSe analyses in the Galaxy LEfSe module (alpha set to 0.01).

### Transcriptome

Flash-frozen rat colon tissues (n = 3–5 replicates per group) were subjected to total RNA extraction using a Qiagen miRNeasy mini kit, according to the manufacturer’s instructions. After DNase digestion and purity/integrity confirmation, as reported,^12,14^ RNA samples were run on a 1% denaturing agarose gel prior to library preparation for RNA and small RNA sequencing. For RNA library preparation, NEBNext® Poly(A) mRNA Magnetic Isolation Module (NEB, E7490) and NEBNext® Ultra™ Directional RNA Library Prep Kit (NEB, E7420) were performed with 3 μg RNA input as the starting material. For small RNA library preparation, NEBNext® Small RNA Library Prep Set for Illumina® (NEB, E7300) were performed with 1 μg RNA input as the starting material. The RNA went through 3ʹ ligation, primer hybridization, 5ʹ ligation, first strand synthesis, PCR amplification and size selection (MinElute Gel Extraction Kit, Qiagen). During library construction, each sample was tagged with a specific index at the PCR amplification step. The library concentration was measured by Qubit dsDNA HS Assay Kit (Thermo Fisher Scientific) and the size of the library was assessed by 2% agarose gel with 2 ng library material. Samples were diluted to 1 pmol for further library pooling. Before loading the pooled library into the NextSeq 550/500 system (Illumina), 1 nM library was denatured and diluted according to NextSeq System Denature and Dilute Libraries Guide (Illumina). Stranded RNA sequencing was conducted by paired-end sequencing with 75-bp read length and small RNA sequencing was conducted by single-end sequencing with 50 bp read length. Fastq files were generated on BaseSpace for further bioinformatics processing and analysis.

### Metabolome

Pre-weighed samples of rat colon tumor and normal colonic mucosa (n = 6–7 matched biological replicates), collected at the time of necropsy, were homogenized in 0.5 ml cold methanol and 0.2 ml chloroform in pre-cooled Garnet bead tubes using a Precellys®24 beadbeater. Samples were centrifuged at 3000 rpm for 10 mins at 4°C and 0.7 ml cold water was added to the supernatant. The aqueous phase was collected by centrifugation at 3000 rpm for 1 min, and the extraction procedure was repeated. The pooled aqueous (upper) phase was passed through a sterile nylon cell strainer and lyophilized (Labconco^TM^). Lyophilized samples were reconstituted in 50 μl methanol/water (1:1, *v/v*) and stored at −80°C until analysis. Untargeted liquid chromatography high-resolution accurate-mass spectrometry (LC-HRAM^TM^) profiling was conducted on a Q Exactive™ Plus Hybrid Quadrupole-Orbitrap™ Mass Spectrometer coupled to a Dionex UltiMate 3000 high-performance liquid chromatography system (Thermo Scientific^TM^). A Synergi Fusion-RP C-18 column (Phenomenex) was used with a methanol/acetonitrile solvent gradient, and mass scanning in the positive mode was in the range 50 to 750. The MS1 and MS1-dependent MS2 spectra were collected at an *m/z* resolution of 70,000 and 17,500, respectively, with the autosampler maintained at 4°C. Methanol/water (1:1 *v/v*) blanks were injected between each run to prevent sample carryover. Parallel unbiased LC-MS studies also were conducted on the freeze-dried spinach, taking multiple random samples of the batch material prior to incorporation into the rodent AIN basal diet. Samples (n = 10) were shipped cold overnight in foil-wrapped sealed containers, and analyzed by Professor Kyongbum Lee, Department of Chemical and Biological Engineering, Tufts University, Medford, MA.

Raw metabolomic data were imported into Progenesis QI (Waters) for alignment, peak picking, and compound identification. Among the 17243 features detected, candidates were identified by reference to the Human Metabolome Database (HMDB) and KEGG. Raw abundance data were normalized to initial sample weights, incorporating Partial Least Squares Discriminant Analysis (PLSDA). Features were further filtered by their appearance in three independent metabolomic databases, with at least three biological replicates and a significant ANOVA test. This resulted in 5946 differential features for further analysis. Significant features were subjected to clustering and correlation by MetaboAnalyst 4.0.^74–77^ The *p*-values (two-tailed *t*-test) and *t*-scores (standardized test statistic) were generated for multiple group comparisons of metabolic networks and functional metabolite prediction via Mummichog version 2 in R.^78^ Primary prediction of 883 compound names was mapped to the KEGG COMPOUND Database, and pathway analyses by Mummichog were ranked according to the *p*-value, using *p* = .05 as the cutoff.

### Bioinformatics

RNA-seq data were processed and analyzed as reported.^12^ DEGs were called using DESeq2, with adjusted *p*-value <0.05. MiRNA-seq raw Fastq data were trimmed using cutadaptor, and reads with the same sequence were collapsed and counted. Mature and hairpin miRNA sequence data for rat were downloaded from miRbase (http://www.mirbase.org/ftp.shtml). Collapsed reads were mapped to rat mature miRNA sequences using blastn. The counts table was input to DESeq2 to call DEmiRs with adjusted *p*-value <0.05. PCA was performed using DESeq2 for both RNA-seq and miRNA-seq datasets. Functional term-enrichment of DEGs used GSEA (Java Desktop v3.0 Beta 2) with FDR cutoff <0.05. Six miRNAs were prioritized for target RNA analysis. Target prediction was conducted by TargetScan 7.2 for rat specific analysis, and miRNA-mRNA pairs with *p*-value < −0.5 were retained in Cytoscape.^79^ Additional methodologies were as reported.^80–83^

### RNA

RNA extraction and purification were as reported, using a minimum of three biological replicates per group.^12–14,84–86^ After SuperScript III (Thermo Fisher Scientific) or miScript RT II (Qiagen) kits, quantitative PCR (qPCR) reactions were performed by LightCycler® FastStart DNA Master SYBR Green (Roche Applied Science) on a LightCycler96 instrument. Primers for mRNA qPCR were designed by NCBI-BLAST (see Supplemental Table S11), whereas primers for miRNA qPCR were purchased custom-made from Qiagen. Internal controls were *Gapdh* and *U6B* small nuclear RNA for mRNA and miRNA analyses, respectively. RNAs and miRNAs were selected based on prior validation and ranking in GSEA data. Original gene lists for tight junction and anti-microbial function were obtained from KEGG and Gene Ontology (GO) resources, respectively, and further sorted by the sequencing data to generate genes of interest. We focused on six miRNAs consistently altered in Pirc colon tumors, and filtered miRNA-mRNA pairs by conserved UTR target site in human and rat, with a linear correlation <-0.7 in sequencing data. Verification of miRNAs and mRNAs was by qPCR (Pearson’s test, with r <-0.5).

### Statistics

Statistical analysis of two-group comparisons was performed using an unpaired two-tailed *t*-test. Correlation analysis was performed by linear and Pearson’s correlation, for miRNA-RNA target and microbiome-host correlations, in tumor and diet intervention groups. For matched tumor outcome-microbiome correlation analysis, seven biological replicates of Pirc/Ctrl or Pirc/SPI were undertaken, whereas three biological replicates were used for SPI responsive gene-microbiome correlations. Unbiased metabolomic analyses typically used n = 6–7 biological replicates per group. In the figures, each datapoint designates a single colon tumor or normal colonic mucosa sample from individual rats in the corresponding groups. One-way ANOVA was used to compare the mean of each column with the mean of every other column, with Tukey correction for multiple comparisons (GraphPad Prism 9.0). The level of significance was designated in the figures as follows: *p < .05; **p < .01; ***p < .001; ****p < .0001, or with the exact *p* value.^85–90^

## Abbreviations

**Table:** 

Apc	Adenomatous polyposis coli
ATIMA	Agile Toolkit for Incisive Microbial Analyses
BrdU	bromodeoxyuridine
CMMR	Center for Metagenomic and Microbiome Research
CRC	colorectal cancer
COX	cyclooxygenases
Ctrl	control
CYP	cytochromes P450
DEGs	differentially expressed genes
DEmiRs	differentially expressed miRNAs
DHET	dihydroxyicosatrienoic acid
8,9-DHET	8,9-dihydroxy-5Z,11Z,14Z-icosatrienoic acid
GO	Gene Ontology
GPx	glutathione peroxidase
GSEA	Gene Set Enrichment Analysis
H&E	hematoxylin and eosin
HETE	hydroxyeicosatetraenoic acid
13-(S)HODE	(13S)-hydroxyoctadecadienoic acid
5-HPETE	5-hydroperoxyeicosatetraenoic acid
KEGG	Kyoto Encyclopedia of Genes and Genomes
6-keto-PGE1	6-keto prostaglandin E1
6-keto-PGF1α	6-keto prostaglandin F1α
9(S)HETE	(19S)-hydroxyeicosatetraenoic acid
13-HPODE	13S-hydroperoxy-9Z,11E-octadecadienoic acid
9(S)-HPOT	(10E,12Z,15Z)-(9S)-9-hydroperoxyoctadeca-10,12,15-trienoic acid
LEfSe	LDA Effect Size
LTA_4_	leukotriene A_4_
miRNA	microRNA
5-LOX	5-lipoxygenase
9-LOX	9-lipoxygenase
15-LOX-1	15-lipoxygenase-1
OTUs	Operational Taxonomic Units
13-oxoODE	(9Z,11E)-13-oxooctadeca-9,11-dienoic acid
PARP	poly (ADP-ribose) polymerase
PCA	Principal Component Analysis
PCoA	Principal Coordinate Analysis
PGE2	prostaglandin E2
PICRUSt	Phylogenetic Investigation of Communities by Reconstruction of Unobserved States
Pirc	polyposis in rat colon
PLA_2_	phospholipase A_2_
RNA-seq	RNA sequencing
RPKM	reads per kilo base per million mapped reads
RT-qPCR	reverse transcription quantitative polymerase chain reaction
SPI	spinach
STAMP	Statistical Analysis of Metagenomic Profiles
WT	wild type.

## Supplementary Material

Supplemental Material

## Data Availability

Data associated with this article were deposited in GEO with accession number GSE180160.

## Author details

^1^Center for Epigenetics & Disease Prevention, Texas A&M Health, Houston, TX, USA; ^2^Deparment of Chemical Engineering, College of Engineering, Texas A&M University, College Station, TX, USA; ^3^Department of Chemical and Biological Engineering, Tufts University, Medford, MA, USA; ^4^Department of Translational Medical Sciences, Texas A&M College of Medicine, Houston, TX, USA.

## Health and Safety

All mandatory laboratory health and safety procedures have been complied with in the course of conducting the experimental work. This included, in the latter stages, following local, state and national guidelines on social distancing, wearing of PPE, staggered work schedules, and other covid-related safety measures.
