# Supplementary material for: Dietary spinach reshapes the gut microbiome in an Apc-mutant genetic background: mechanistic insights from integrated multi-omics
Source: Gut Microbes. 2021 Sep 8;13(1):1972756. doi: 10.1080/19490976.2021.1972756 (PMC8437542; doi:10.1080/19490976.2021.1972756)
Supplement: Supplemental Material [file KGMI_A_1972756_SM1233.zip › Supplementary information/YS Chen Supplemental Figure 1.pptx]

## Slide 1
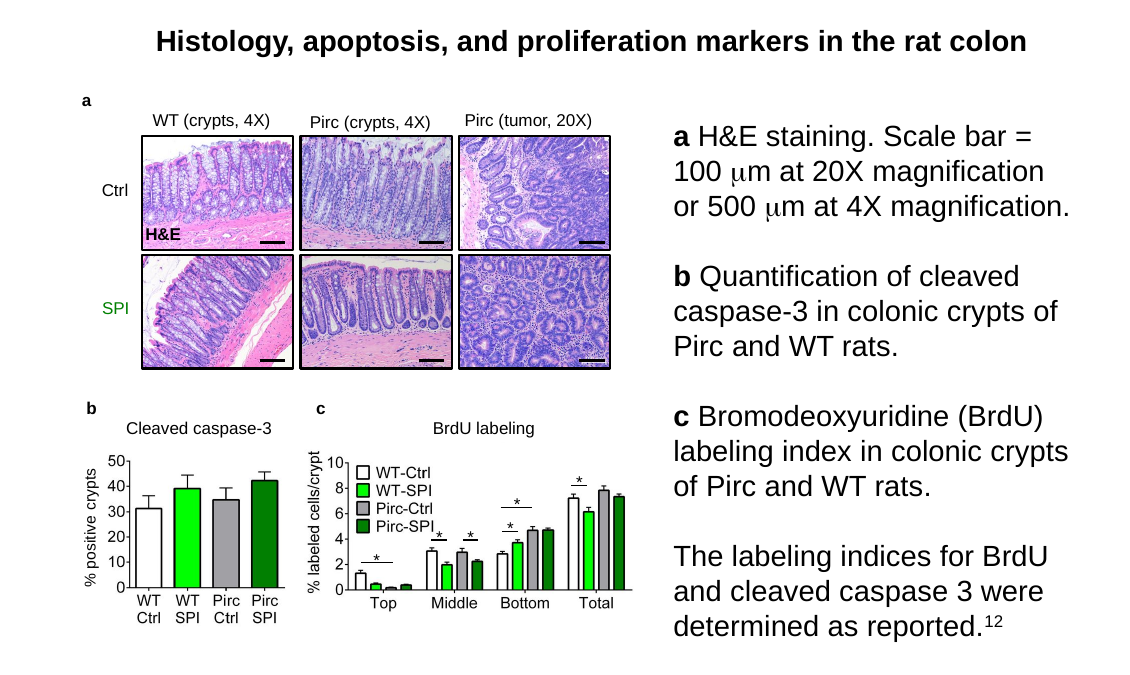

Histology, apoptosis, and proliferation markers in the rat colon
a
WT (crypts, 4X)
Pirc (tumor, 20X)
Pirc (crypts, 4X)
Ctrl
H&E
SPI
a H&E staining. Scale bar = 100 mm at 20X magnification or 500 mm at 4X magnification.
b Quantification of cleaved caspase-3 in colonic crypts of Pirc and WT rats.
c Bromodeoxyuridine (BrdU) labeling index in colonic crypts of Pirc and WT rats.
The labeling indices for BrdU and cleaved caspase 3 were determined as reported.12
b
c
Cleaved caspase-3
BrdU labeling
*
*
% positive crypts
*
*
*
*
