# Supplementary material for: Dietary spinach reshapes the gut microbiome in an Apc-mutant genetic background: mechanistic insights from integrated multi-omics
Source: Gut Microbes. 2021 Sep 8;13(1):1972756. doi: 10.1080/19490976.2021.1972756 (PMC8437542; doi:10.1080/19490976.2021.1972756)
Supplement: Supplemental Material [file KGMI_A_1972756_SM1233.zip › Supplementary information/YS Chen Supplemental Figure 2.pptx]

## Slide 1
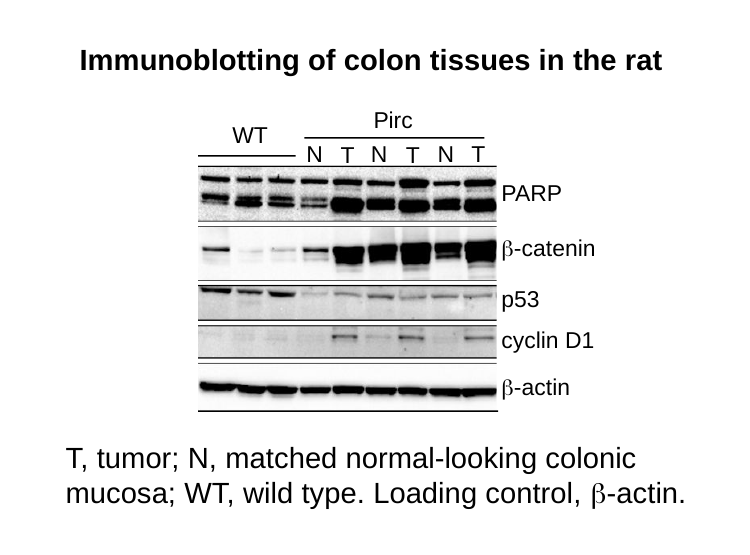

Immunoblotting of colon tissues in the rat
Pirc
WT
N
N
N
T
T
T
PARP
b-catenin
p53
cyclin D1
b-actin
T, tumor; N, matched normal-looking colonic mucosa; WT, wild type. Loading control, b-actin.
