# Supplementary material for: Dietary spinach reshapes the gut microbiome in an Apc-mutant genetic background: mechanistic insights from integrated multi-omics
Source: Gut Microbes. 2021 Sep 8;13(1):1972756. doi: 10.1080/19490976.2021.1972756 (PMC8437542; doi:10.1080/19490976.2021.1972756)
Supplement: Supplemental Material [file KGMI_A_1972756_SM1233.zip › Supplementary information/YS Chen Supplemental Figure 3 REVISED.pptx]

## Slide 1
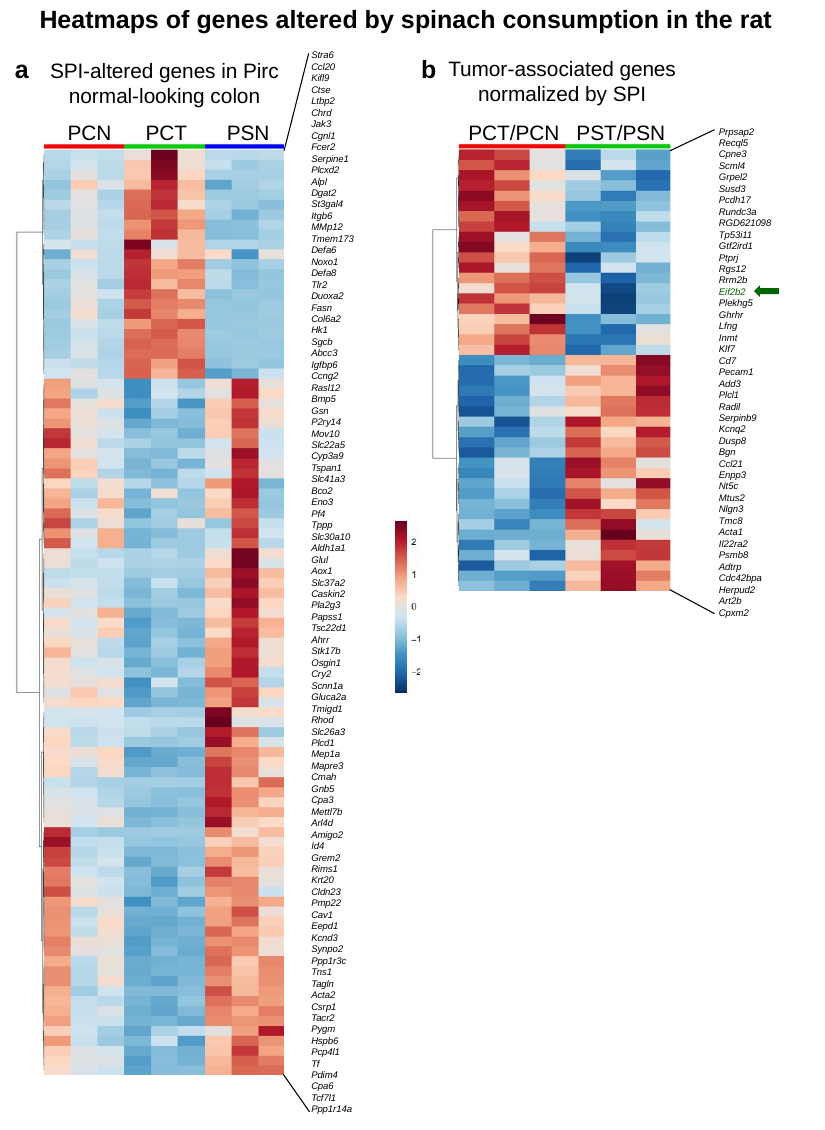

Heatmaps of genes altered by spinach consumption in the rat
b
a
Tumor-associated genes normalized by SPI
Stra6
Ccl20
Kifl9
Ctse
Ltbp2
Chrd
Jak3
Cgnl1
Fcer2
Serpine1
Plcxd2
Alpl
Dgat2
St3gal4
Itgb6
MMp12
Tmem173
Defa6
Noxo1
Defa8
Tlr2
Duoxa2
Fasn
Col6a2
Hk1
Sgcb
Abcc3
Igfbp6
Ccng2
Rasl12
Bmp5
Gsn
P2ry14
Mov10
Slc22a5
Cyp3a9
Tspan1
Slc41a3
Bco2
Eno3
Pf4
Tppp
Slc30a10
Aldh1a1
Glul
Aox1
Slc37a2
Caskin2
Pla2g3
Papss1
Tsc22d1
Ahrr
Stk17b
Osgin1
Cry2
Scnn1a
Gluca2a
Tmigd1
Rhod
Slc26a3
Plcd1
Mep1a
Mapre3
Cmah
Gnb5
Cpa3
Mettl7b
Arl4d
Amigo2
Id4
Grem2
Rims1
Krt20
Cldn23
Pmp22
Cav1
Eepd1
Kcnd3
Synpo2
Ppp1r3c
Tns1
Tagln
Acta2
Csrp1
Tacr2
Pygm
Hspb6
Pcp4l1
Tf
Pdim4
Cpa6
Tcf7l1
Ppp1r14a
SPI-altered genes in Pirc normal-looking colon
PCN PCT PSN
PCT/PCN PST/PSN
Prpsap2
Recql5
Cpne3
Scml4
Grpel2
Susd3
Pcdh17
Rundc3a
RGD621098
Tp53i11
Gtf2ird1
Ptprj
Rgs12
Rrm2b
Eif2b2
Plekhg5
Ghrhr
Lfng
Inmt
Klf7
Cd7
Pecam1
Add3
Plcl1
Radil
Serpinb9
Kcnq2
Dusp8
Bgn
Ccl21
Enpp3
Nt5c
Mtus2
Nlgn3
Tmc8
Acta1
Il22ra2
Psmb8
Adtrp
Cdc42bpa
Herpud2
Art2b
Cpxm2
