# Supplementary material for: Dietary spinach reshapes the gut microbiome in an Apc-mutant genetic background: mechanistic insights from integrated multi-omics
Source: Gut Microbes. 2021 Sep 8;13(1):1972756. doi: 10.1080/19490976.2021.1972756 (PMC8437542; doi:10.1080/19490976.2021.1972756)
Supplement: Supplemental Material [file KGMI_A_1972756_SM1233.zip › Supplementary information/YS Chen Supplemental Figure 4.pptx]

## Slide 1
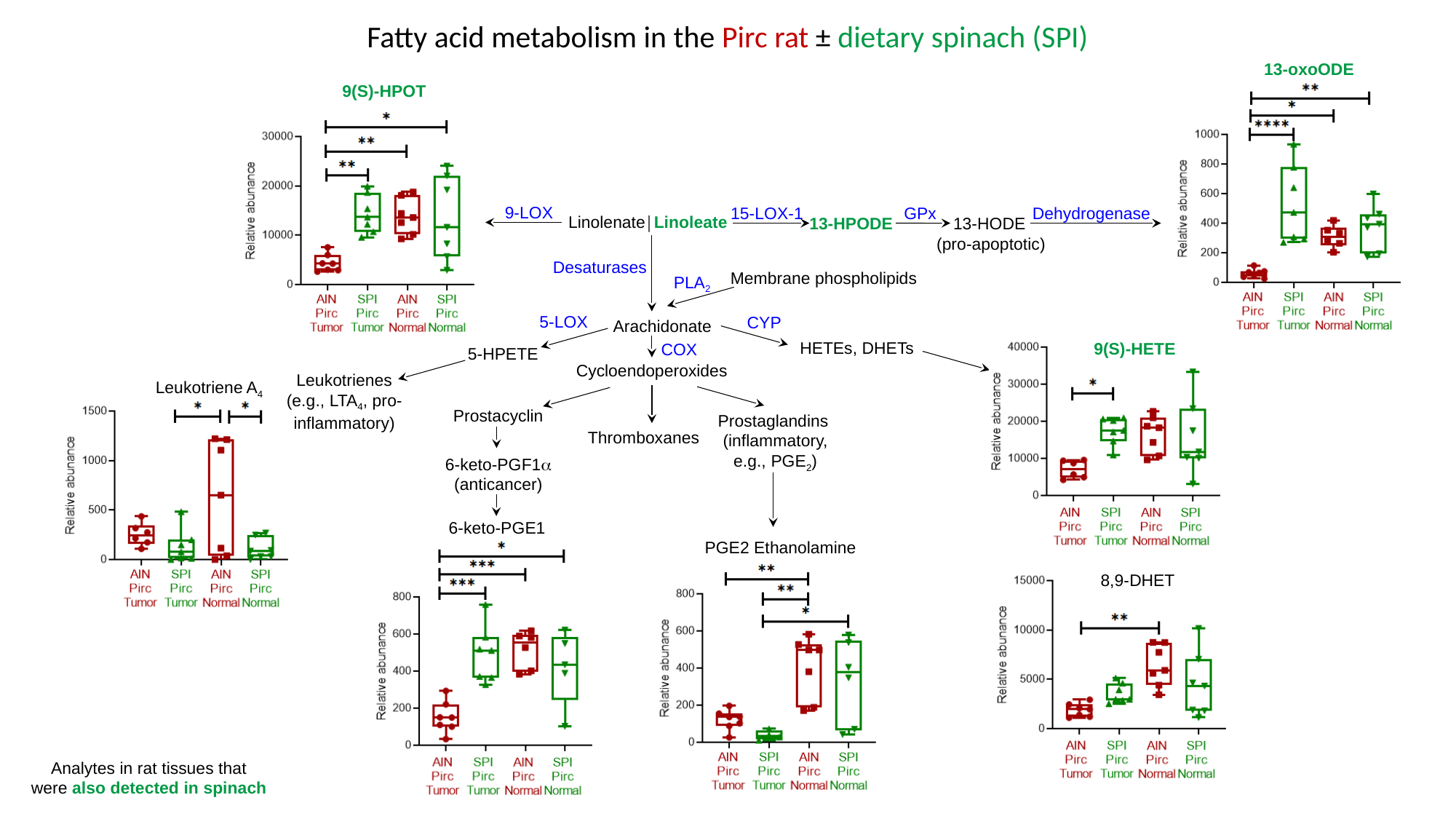

Fatty acid metabolism in the Pirc rat ± dietary spinach (SPI)
13-oxoODE
9(S)-HPOT
9-LOX
15-LOX-1
GPx
Dehydrogenase
Linolenate│Linoleate
13-HPODE
13-HODE
(pro-apoptotic)
Desaturases
Membrane phospholipids
PLA2
5-LOX
CYP
Arachidonate
HETEs, DHETs
9(S)-HETE
COX
5-HPETE
Leukotriene A4
Cycloendoperoxides
Leukotrienes (e.g., LTA4, pro-inflammatory)
Prostacyclin
Prostaglandins (inflammatory, e.g., PGE2)
Thromboxanes
6-keto-PGF1a (anticancer)
6-keto-PGE1
PGE2 Ethanolamine
8,9-DHET
Analytes in rat tissues that were also detected in spinach
